# Falls and Sleep Disorders in Spanish Alzheimer’s Disease in Nursing Homes: An Observational Study

**DOI:** 10.3390/healthcare11212852

**Published:** 2023-10-30

**Authors:** Rubén Cámara-Calmaestra, Antonio Martínez-Amat, Agustín Aibar-Almazán, Fidel Hita-Contreras, Nerea De Miguel-Hernando, Daniel Rodríguez-Almagro, Raquel Fábrega-Cuadros, Alexander Achalandabaso-Ochoa

**Affiliations:** 1 Department of Health Sciences, Faculty of Health Sciences, University of Jaén, 23071 Jaén, Spainrfabrega@ujaen.es (R.F.-C.);; 2 Department of Surgery, Ophthalmology, Otolaryngology and Physiotherapy, University of Valladolid, 47002 Valladolid, Spain; 3 Nursing, Physical Therapy and Medicine, University of Almería, 04120 Almería, Spain; dra243@ual.es

**Keywords:** risk of falls, Alzheimer, type II diabetes mellitus, cross-sectional

## Abstract

Objective: The main objective of this study was to establish a relationship between the number of falls and sleep problems experienced by patients with Alzheimer’s disease. Materials and Methods: This was a cross-sectional study. A total of 114 Spanish aged people with Alzheimer’s disease institutionalized in nursing homes and 80 independent Spanish aged people without neurodegenerative diseases living at home were enrolled in this study and completed in-person interviews and digital questionnaires. Results: The mean age was 78.98 ± 8.59 years. Sleep disorders were related to continuous stress (*p* = 0.001; OR = 4.729) and a high frequency of falls (*p* = 0.001; OR = 2.145), while predictor variables associated with falls in patients with Alzheimer’s disease were continuous medical visits (β = 0.319, *p* < 0.001), family history of dementia (β = 0.212; *p* = 0.014), and sleep disorders (β = 0.235; *p* = 0.007). Second, the analysis showed that moderate physical activity (*p* = 0.001; OR = 0.147), continuous medical visits (*p* < 0.001; OR = 0.621), and high level of study (*p* = 0.011; OR = 0.334) were protective factors against Alzheimer’s, while older age (*p* = 0.035; OR = 1.087), type II Diabetes Mellitus (*p* = 0.042; OR = 3.973), number of falls (*p* = 0.021; OR = 1.409), and daily drug intake (*p* = 0.001; OR = 1.437) were risk factors for Alzheimer’s. Conclusions: Sleep disturbances are related to stress and falls in a sample of 114 Spanish AD aged people institutionalized in nursing homes, and the falls they experience are related to ongoing medical visits, a history of dementia, and sleep disturbances. Therefore, a bidirectional relationship was established between falls and sleep disorders in these patients. Moreover, this study showed that a greater frequency of falls and high daily drug intake could constitute novel risk factors for Alzheimer’s disease, in addition to already known factors, such as age and type II Diabetes Mellitus, while being physically active and a high level of studies are protective factors against Alzheimer’s disease.

## 1. Introduction

Alzheimer’s disease (AD) is the most common neurodegenerative disease and the most frequent cause of dementia [1,2]. The main hallmarks of this disease are intraneuronal neurofibrillary tangles and extracellular deposits (plaques) of amyloid proteins. In addition to the multiple cognitive symptoms presented by AD, motor symptoms manifest during the early stages of the disease [3]. Recently, it was observed that the binomial formed by cognitive and motor symptoms produced by AD contributes to gait alterations and increases the risk of falling in individuals with this disorder [3]. Alzheimer’s patients showed a significantly higher risk of falling than non-demented patients, and this risk has been observed to be up to 10 times higher [4]. Moreover, a fall in AD may result in more serious and costly injuries [5]. In addition to an increased risk of falls, individuals with AD also experience a greater proportion of sleep alterations, which can appear prior to cognitive decline [6]. Furthermore, current evidence indicates that sleep disorders could be related to AD pathogenesis, and brain areas affected by sleep disorders coincide with vulnerable areas in AD [7]. Therefore, due to the high prevalence and incidence of AD in the aging world population [8], the impact of falls and sleep disorders will be greater in the coming years. However, the relationship between these variables in these patients has not been studied.

On the other hand, many studies have reported which modifiable factors increase the risk of AD or protect against its appearance to reduce its incidence and the high costs this disease inflicts on the social health system [9]. In this way, some previous studies have already identified some risk AD factors, such as mid-life cardiovascular diseases, type II Diabetes Mellitus, sleep disorders, obesity, depression, stress, hearing loss, and oral diseases, while higher education level, physical activity, Mediterranean diet, bilingualism, and social engagement have been reported to be protective factors for AD [10,11]. In addition, considering that AD begins decades before clinical symptoms occur, identifying risk and protective factors against it could prevent or delay the onset of the disease in older people without dementia and even in the middle-aged population [12].

Despite current evidence indicating that sleep disorders represent an AD risk factor, in addition to being more frequently experienced in these patients, and considering the enormous problems that falls generate in this population, there are no previous studies that have related these variables. Therefore, this study could fill this gap by shedding light on how falls and sleep problems are linked in individuals with AD. Hence, there is a need for an observational study, the main objective of which is to establish relationships between falls and sleep problems experienced by patients with AD. As a secondary objective, it was proposed to identify any other possible risk and protective factors related to AD to update previous literature in this field and provide some AD preventive future interventions.

## 2. Materials and Methods

### 2.1. Study Design

A cross-sectional study was conducted, whose design and drafting were carried out according to the statement “Strengthening the reporting of observational studies in epidemiology” guide (STROBE) to accurately describe observational studies [13].

### 2.2. Background

This study was developed in different cities of Andalusia, Spain. Specifically, data were collected from participants living in these cities: Algeciras, Andújar, Jaén, Torredonjimeno, and Úbeda. The recruitment and data collection period took place from 1 April 2021 to 31 May 2022.

### 2.3. Participants

The participants provided their consent to participate physically or digitally using the same informed consent model. In the event that the patient was incapacitated, authorization from a family member in charge of the patient was required.

The selection of the sample was carried out through an interview or online questionnaire with all participants interested in being part of the study. A total of 114 Spanish aged people with Alzheimer’s disease institutionalized in nursing homes and 80 independent Spanish aged people without neurodegenerative diseases living at home were enrolled in this study. As for the Alzheimer’s sample, 110 patients could fill out the survey by themselves, while the remaining 4 needed a family member to fill out the survey.

For the Alzheimer’s sample, 18 nursing homes specialized in Alzheimer’s from different parts of the geography of Andalusia were contacted, of which 4 answered affirmatively. For participants without neurodegenerative diseases, only the online survey was disseminated through social networks (available in Supplementary Materials).

The eligibility criteria for the Alzheimer’s sample were as follows: (i) age ≥ 65 years; (ii) medical diagnosis of probable Alzheimer’s disease by a neurologist, according to the National Institute on Aging–Alzheimer’s Association workgroups on diagnostic guidelines for Alzheimer’s disease [14]; and (iii) informed consent in physical or digital format, of the participant himself or by an authorized relative, in case the participant was incapacitated. In patients without neurodegenerative diseases, the following eligibility criteria were used: (i) age ≥ 65 years; (ii) not suffering from and/or diagnosed with any neurodegenerative disease; and (iii) independent living at home.

On the other hand, the exclusion criteria for both samples were as follows: (i) failure to duly complete informed consent and (ii) impossibility of collecting any of the observed variables.

**Figure 1:**
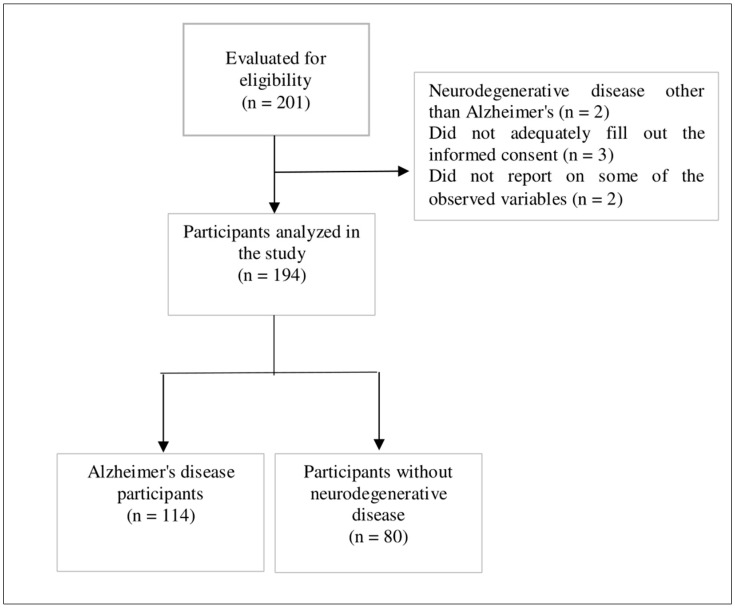
Flow diagram of the study participants.

The same survey was used in a physical or digital format to obtain sociodemographic data (age, sex, place of residence, marital status) and information related to the other variables of interest. All data were collected in an orderly manner using Excel. The participant selection process is illustrated (Figure 1).

#### 2.3.1. Variables and Data Sources

The variables were collected through online surveys or in-person interviews. Quantitative variables collected were age, number of falls, medical visits in the last year, daily drug intake, stature, weight, height, and Body Mass Index (BMI). For the weight, this variable was collected numerically and expressed in kilograms. In nursing homes, a digital scale Tanita HD-318^®^ (Tanita, Tokyo, Japan) was used to measure weight and BMI, which has valid and reliable results (ICC intra- and inter-examiner = 0.9990) [15].

Qualitative variables collected were sex, place of residence, marital status, profession, education level, economic level, diet, attendance at a day center, family history of dementia, physical activity level and toxic habits throughout life, need for a caregiver throughout the day, psychiatric and sleep problems, and work/family stress level throughout life. Toxic habits were collected by asking if the patient had a usual intake of alcohol, tobacco, or drugs during their life; for diet, patients answered if they thought they had a healthy diet or not during their life; and for sleep problems, they were asked if they presented problems such as insomnia, excessive sleepiness, sleep apnea, or fractionated sleep throughout life. The variable physical activity level was collected according to whether a minimum of 150 min per week of moderate non-work physical activity had been carried out throughout their life, which is considered to be physically active [16]. The other variables described above were collected through a bicategorical response. In addition, the medical history was recorded using a national health system medical history document.

#### 2.3.2. Bias

To reduce information bias from medical data, all participants were asked to submit an updated sheet of medical history provided by the national health system. This information was collected in a digital folder and encrypted using a key known only by the principal investigators. The physical documentation was then destroyed.

#### 2.3.3. Sample Size

Sample size calculation was carried out following the criteria established by Calvo et al. [17], where a minimum of ten observations of each variable were introduced in the multiple logistic regression model; likewise, respecting the recommendations of Concato et al. [18], it was necessary for ten observations per variable in the multiple linear regression model. Considering that a maximum of 11 variables were considered for being introduced in the multiple logistic regression model, and an estimated Alzheimer’s disease prevalence of around 60%, a minimum of 183 subjects was necessary for our study purpose.

#### 2.3.4. Statistical Analysis

Data were managed and analyzed using IBM SPSS Statistics 23.0 for Windows (SPSS Inc., Chicago, IL, USA). For the data description of continuous variables, means and standard deviations were employed, while frequencies and percentages were used for categorical variables. Furthermore, normality and homoscedasticity of continuous variables were determined using Kolmogorov–Smirnov and Levene’s tests, respectively, and the chi-squared test and Student’s *t*-test were employed to evaluate differences between groups for categorical and continuous variables, respectively.

To identify the factors related to Alzheimer’s disease, a multiple logistic regression model was carried out over the whole sample, as the cause of the dichotomous nature of the dependent variable. An individualized simple logistic regression model was used to analyze the relationship between the dependent (participants with Alzheimer’s disease/participants without neurodegenerative disease) and independent variables, where each independent variable that showed a statistically significant odds ratio value (*p* < 0.05) was included in the multiple logistic regression model through the introduction method.

In the same mode, and following the same procedure, a multiple logistic regression model was performed to determine the factors related to sleep disturbances in subjects with Alzheimer’s disease, introducing into the multiple model all independent variables that showed statistically significant odds ratios (*p* < 0.05) in the analysis of the relationships between the dependent (sleep disturbances) and independent variables. This analysis was carried out employing only the data from Alzheimer’s disease subjects.

As a cause of the continuous nature of the dependent variable, factors related to the number of falls in subjects with Alzheimer’s disease were predicted through a multiple linear regression model employing only the data from Alzheimer’s disease subjects. A specific simple linear regression model was used to analyze each relationship between the dependent (number of falls) and independent variables, where each independent variable that showed a statistically significant beta value (*p* < 0.05) was included in the multiple linear regression model using the introduction method.

The effect size for the logistic regression model was set using Nagelkerke’s R^2^, whereas the multivariate coefficient of determination (R^2^) was employed for the linear regression models. Following Cohen’s criteria [19], values of R^2^ lower than 0.02 would be considered insignificant effect sizes, between 0.02 and 0.15 would be considered small, between 0.15 and 0.35 medium values, and large above 0.35. Statistical significance was set at *p*-value < 0.05.

## 3. Results

In the present study, 194 subjects were evaluated, of which 114 were subjects with Alzheimer’s disease and the rest were healthy subjects, with a mean age of 78.98 years old (SD = 8.59). The female population comprised two-thirds of the sample. Moreover, remarkable differences in study and economic level, physical activity, healthy diet, and Diabetes Mellitus were observed between healthy and Alzheimer’s subjects, where healthy subjects presented higher study, economic, and physical activity levels than Alzheimer’s subjects, and subjects with Alzheimer’s disease showed higher Diabetes Mellitus levels than healthy subjects (Table 1).

Multiple logistic regression was performed to identify the factors related to Alzheimer’s and predicted 76.8% of the variance of the dependent variable (R^2^ = 0.768; *p* < 0.001). The analysis showed that performing physical activity for at least 150 min per week (*p* = 0.001; OR = 0.147), visiting the physician with high frequency (*p* < 0.001; OR = 0.621), and having a high study level (*p* = 0.011; OR = 0.334) were protective factors, while high age, Diabetes Mellitus, number of falls, and number of drugs were risk factors for the appearance of Alzheimer’s disease (Table 2).

Multiple logistic regression was performed to identify the factors related to sleep disturbances in subjects with Alzheimer’s and predicted 34.8% of the variance in the dependent variable (R^2^ = 0.348; *p* < 0.001). The analysis showed that ongoing stress was a risk factor for sleep disturbances in patients with Alzheimer’s disease (OR = 4.729; *p* = 0.001), as well as a high frequency of falls (OR = 2.145; *p* = 0.001) (Table 3).

Multiple linear regression was performed to identify the factors related to the number of falls in subjects with Alzheimer’s and predicted 21.6% of the variance in the dependent variable (R^2^ = 0.216; *p* < 0.001). The analysis showed that the predictor variables related to the number of falls in patients with Alzheimer’s disease were visiting the physician at a high frequency (β = 0.319; *p* < 0.001), history of dementia (β = 0.212; *p* = 0.014), and sleep disturbances (β = 0.235; *p* = 0.007) (Table 4).

## 4. Discussion

Our results showed sleep problems experienced by patients with this disease seem to be related to stress and falls. The number of falls experienced by Alzheimer’s patients seems to be related to frequent visits to the doctor, family history of dementia, and sleep problems. Age, type II Diabetes Mellitus, number of falls, and high daily drug consumption have been identified as risk factors for the onset of Alzheimer’s disease. On the contrary, it has been observed that being physically active, visiting a doctor regularly, and having a high level of studies seem to have a protective effect against the onset of Alzheimer’s disease.

Next, our starting issue will be the falls suffered by patients with Alzheimer’s disease, which are related to frequent visits to the doctor, family history of dementia, and sleep problems.

In relation to medical visits, some studies showed that Alzheimer’s patients produce a higher health expenditure, not only at medical visits, but also in costs for institutionalization and hospitalization [20]. The greater frequency of medical visits is associated with illness and frailty, both factors related to an increased risk of falls [21]. In addition, this age group already had a greater number of medical visits [22]. These data suggest an association between visiting the doctor more frequently and an increased risk of falling in patients with AD.

Continuing to a family history of dementia, it has been previously described that patients with Alzheimer’s disease have a higher risk of falling; adding the possible genetic load that predisposes them via a family history of dementia, this could lead to an even greater risk of falls, as our results showed. Our data agree with previous scientific literature stating there is a direct relationship between greater cognitive impairment and a greater risk of falling [23]. Moreover, a study showed that relatives of those with dementia presented worse cognitive performance than individuals without a genetic background [24], especially those with a parental history of dementia. Therefore, these populations could have more severe cognitive impairment, which could explain the increased risk of falls.

Finally, sleep problems have also been linked to an increased risk of falls in patients with AD, which seems to be bidirectional, as shown by the results of our study. One explanation could be that sleep disorders can cause a greater accumulation of tau protein and amyloid beta [25], which are markers of the disease par excellence, further exacerbating these sleep disorders [26]. These are associated with a decrease in cognitive performance in several domains, such as the ability to concentrate, memory, and attention problems, which have already been associated with an increased risk of falls [27]. On the other hand, evidence has shown that the risk of falls could increase fear of falling [28], and a greater fear of falling is related to greater cognitive decline in the aged population [29], even more so in patients with Alzheimer’s disease. Moreover, cognitive decline has been related to sleep disorders in the Alzheimer’s population, as mentioned in a recent meta-analysis [30].

Therefore, it seems undeniable that tools aimed at reducing the risk of falls, sleep disorders, and cognitive decline in this population that have mechanisms of action both physically and cognitively are needed, as physical exercise has already been shown in patients with Alzheimer’s disease [5,31,32].

Consistent with our study results, continuous stress has also been linked to sleep disorders in patients with AD. This relationship is widely described in the scientific literature, since the body prepares to deal with stressful situations by releasing hormones, such as cortisol, which activate our state of alertness and threat in order to cope with these situations. If these states are maintained continuously, the regeneration processes that occur in the body cannot be carried out, affecting the physical–cognitive level and causing exhaustion and triggering processes that alter homeostasis. These continuous stress states activate pathways contrary to those needed when sleeping, depriving us of the repair processes associated with sleep [33,34]. In addition, continued stress causes damage to the hippocampus and other parts of the brain [35]. Since Alzheimer’s patients experience prolonged stress situations due to cognitive problems, such as confusion and interpretative errors, and a multitude of neuropsychiatric symptoms may appear, this stress damage could severely impact sleep.

Hence, it could be useful to try to deprive Alzheimer’s patients of continuous stress states to reduce the increased sleep disturbances that occur in these patients.

Taking account of the information above, we could hypothesize if suffering more falls, having family history of dementia, and high stress levels would lead to a more severe AD than in people without these features, in the case of suffering from this disease.

Next, we must mention the risk factors that have been related to Alzheimer’s disease, including age, type II Diabetes Mellitus, a greater number of falls, and a greater consumption of drugs.

In relation to age, aging can be considered the most important risk factor for Alzheimer’s disease, since in the vast majority of cases, the disease has a late onset, leading from the age of 65 [36]. Aging is a complex and irreversible process that occurs in multiple organs and cellular systems and is characterized by a decrease in brain weight and volume, loss of synapses, and ventricular widening in specific areas, which are accompanied by deposits of amyloid beta and neurofibrillary tangles via hyperphosphorylation of tau protein, which can produce cognitive impairment, both of which are characteristic Alzheimer’s disease biomarkers [37]. In addition, several studies have already observed the incidence of Alzheimer’s disease as age increases, showing that the upward trend has this binomial [38].

Regarding type II Diabetes Mellitus, there is a molecular relationship between the amyloid polypeptide of the pancreatic islets typical of those who suffer from this type of diabetes and amyloid beta, which could exacerbate cognitive impairment and, therefore, Alzheimer’s disease [39]. In addition, links have already been established between suffering from type II Diabetes Mellitus and having a lower speed of perception and verbal skills [40], which reaffirms the relationship between both pathologies. This relationship has already been observed in numerous studies where type II Diabetes Mellitus is an important risk factor for the onset of Alzheimer’s disease [41].

Considering the greater number of falls and suffering from Alzheimer’s disease, we must consider that falls in the elderly are related to the degree of cognitive impairment, which is also closely linked to gait and balance problems [42]. Therefore, it is possible that individuals who fall the most have poorer cognitive performance and are more susceptible to Alzheimer’s disease in the future. In addition, the fear of falling and isolation produced by falls in older people [43] could be factors that lead to the physical–cognitive deterioration that occurs in these individuals, increasing the risk of suffering from the disease. To our knowledge, no previous studies have reported this association; therefore, our study suggests that the number of falls could constitute a valuable novel variable to study as a risk factor for Alzheimer’s disease.

As the last risk factor in our study, increased drug consumption was linked to AD onset. Previous evidence has indicated that high consumption of drugs can lead to interactions among them and possible adverse reactions [44]. In fact, some studies have examined the association between polypharmacy (consuming five or more drugs daily) and cognitive [45] and functional dysfunction [46]. In addition, current drugs, such as anticholinergics, have been shown to have adverse effects on cognition and have been considered a risk factor for developing Alzheimer’s disease [47]. This physical–cognitive deterioration produced by the high intake of medication could expose older people to a greater extent to Alzheimer’s disease in the future. Moreover, one study hypothesized that polypharmacy could be related to the severity of Alzheimer’s [48], but not to Alzheimer’s onset. Therefore, to the best of our knowledge, our study results suggest that high daily drug intake may be a novel risk factor for the onset of AD.

As recommended, exhaustive and periodic medication control is required, especially in aged people at risk of suffering from Alzheimer’s, in order to avoid polypharmacy and certain types of drugs that could negatively impact their health.

Finally, our study results showed that protective factors against Alzheimer’s disease were physical activity, a high level of education, and regular doctor visits.

As previously observed, low levels of both physical and cognitive abilities can increase the risk of Alzheimer’s disease; therefore, physical exercise is considered key in preventing it. In fact, between 20.3 and 21.8% of the world’s cases of Alzheimer’s are attributed solely to physical inactivity [49], and recent evidence suggests that the protective effect of exercise against Alzheimer’s risk is dose-dependent [50]. Physical exercise can reduce the number of falls and improve cognitive performance and physical functional capacity, and is the only agent that acts at all levels [51,52,53]. This is partly due to the fact that physical exercise can act by increasing the volume of the hippocampus and the level of cerebral perfusion [54,55], in addition to increasing the levels of brain-derived neurotrophic factor (BDNF) [56], which is a growth factor associated with the development and survival of neurons and synapse processes. Moreover, it also increases the elimination of tau protein and amyloid beta [57]. Regarding physical functional capacity, several studies have already declared the effects that physical exercise can provide on it [58,59], and there is even scientific literature that reports an inverse relationship between muscle strength and risk of Alzheimer’s disease [60].

Therefore, it is considered very important to remain physically active throughout life, since physical exercise is a unique tool capable of producing protective effects against Alzheimer’s disease due to its pathways of action.

On the other hand, previous evidence has indicated that around 19.1% of global cases of Alzheimer’s could be due to a low level of education [49], remarking the importance of education in AD. The fact of having a high level of education is related to the formation of greater cognitive reserve and greater brain volume [61]. Cognitive reserve refers to the ability or tolerance that the brain develops to cope with the physiological changes related to aging or some pathology in the absence of clinical symptoms [62]; for this reason, individuals with a high level of education will have a lower risk of developing Alzheimer’s disease. In this line, there is scientific literature indicating that people with a lower level of education and work requirements had an approximately two times higher risk of developing dementia [63], and another study that analyzed more than 70,000 patients with Alzheimer’s showed that about 70% of them had a low education level [46], which is in agreement with our study, where about 93% of participants with Alzheimer’s had a low level of education.

Hence, it is necessary to raise awareness among the population about the importance of having a higher level of education and regularly participating in cognitive activities to protect against the risk of Alzheimer’s disease.

Finally, regular visits to the doctor were linked to protective factors against Alzheimer’s disease. This was surprising, since none of the studies published in this line have reported this relationship, and the number of medical visits is usually related to health problems. This association may be due to the fact that during the study we were in the coronavirus pandemic; therefore, these data could be biased, since the reason for the medical visit was not observed, and the number of medical visits due to the virus in this population group was especially high, either by telephone or in person [22], while individuals with Alzheimer’s were institutionalized, and these medical visits were probably not accounted for due to nursing homes’ medical services covering it. Therefore, despite the results obtained, we cannot guarantee this protective relationship between visiting the doctor and suffering from Alzheimer’s disease, so the need for new studies was detected where this relationship is deepened.

### Limitation

Our study has some limitations. First, the sample size was small compared to similar studies that used a larger sample size. Secondly, some of the variables, such as depression, anxiety, stress, sleep problems, physical activity, or having a healthy diet, were collected by bicategorical response, not using more objective methods for this, such as validated questionnaires for each variable observed. Moreover, the level of cognitive impairment in AD patients was not collected, which would have been interesting for the analysis and establishing relationships with the more affected domains of AD patients. In addition, reasons for medical consultation were not observed, only the number, which could constitute an information bias. Finally, we could not ensure that our healthy sample did not suffer from any neurodegenerative disorder, despite being independent and capable of living in their homes and providing an updated medical history free of neurodegenerative disease.

## 5. Conclusions

Our study showed that sleep disturbances were related to stress and falls in a sample of 114 elderly people with Alzheimer’s disease institutionalized in nursing homes, and the falls they experienced were related to ongoing medical visits, a history of dementia, and sleep disturbances. Therefore, a bidirectional relationship was established between falls and sleep disorders in these patients.

Moreover, this study showed that a greater frequency of falls and high daily drug intake could constitute novel risk factors for Alzheimer’s, in addition to already known factors, such as age and type II Diabetes Mellitus, while being physically active and a high level of studies are protective factors against Alzheimer’s.

As general recommendations in patients with Alzheimer’s disease, regular exercise is necessary to reduce the risk of falls, sleep disorders, and cognitive impairment. In addition, it is recommended to avoid stressful situations to reduce sleep disorders.

As general tips in the non-Alzheimer’s population, it is advisable to practice exercise and have a high level of studies and participation in cognitive tasks to protect against the risk of Alzheimer’s disease. Moreover, exhaustive and periodic medication controls are recommended to avoid polypharmacy, and other drugs may produce adverse health events.

## Figures and Tables

**Table 1:** Morphologic and clinical characteristic of the sample.

Categorical		All (194)		Alzheimer’s (114)		Health (80)		*p*
		Frequency	%	Frequency	%	Frequency	%	
Sex	Female	131	67.53	86	75.44	45	56.25	0.008
	Male	63	32.47	28	24.56	35	43.75	
Study level	None	47	24.23	42	36.84	5	6.25	0.000
	School	100	51.55	65	57.02	35	43.75	
	High school	24	12.37	6	5.26	18	22.50	
	University	23	11.86	1	0.88	22	27.50	
Economic level	Low	31	15.98	26	22.81	5	6.25	0.001
	Medium	149	76.80	77	67.54	72	90.00	
	High	14	7.22	11	9.65	3	3.75	
Diabetes mellitus	No	148	76.29	78	68.42	70	87.50	0.003
	Yes	46	23.71	36	31.58	10	12.50	
Cardiovascular disease	No	86	44.33	44	38.60	42	52.50	0.056
	Yes	108	55.67	70	61.40	38	47.50	
Anxiety	No	186	95.88	111	97.37	75	93.75	0.276
	Yes	8	4.12	3	2.63	5	6.25	
Depression	No	166	85.57	93	81.58	73	91.25	0.095
	Yes	28	14.43	21	18.42	7	8.75	
History of dementia	No	109	56.19	58	50.88	51	63.75	0.076
	Yes	85	43.81	56	49.12	29	36.25	
Physical activity	No	111	57.22	91	79.82	20	25.00	0.000
	Yes	83	42.78	23	20.18	60	75.00	
Sleep disturbances	No	125	64.43	68	59.65	57	71.25	0.128
	Yes	69	35.57	46	40.35	23	28.75	
Healthy diet	No	72	37.11	56	49.12	16	20.00	0.000
	Yes	122	62.89	58	50.88	64	80.00	
Continuous		Mean	Sd	Mean	Sd	Mean	Sd	*p*
Age		78.979	8.592	82.894	7.219	73.450	7.256	<0.001
Weight		66.007	13.954	60.728	12.115	73.529	12.970	<0.001
Height		1.600	0.097	1.566	0.093	1.649	0.082	<0.001
BMI		25.645	4.378	24.731	4.548	26.949	3.785	<0.001
Number of falls in the last year		1.000	1.500	1.307	1.683	0.563	1.054	0.001
Number of visits to physician		3.299	3.642	2.439	1.905	4.525	4.963	0.001
Number of drugs		4.588	3.411	5.825	3.235	2.825	2.845	0.001

Abbreviations: %: percentage; *p*: *p*-value; SD: Standard Deviation; BMI: Body Mass Index.

**Table 2:** Univariate and multivariate logistic regression to analyze the factors related to Alzheimer’s disease.

Variable	OR	Univariate Analysis		*p*	OR	Multivariate Analysis		*p*
		95% C.I.				95% C.I.		
		Inferior	Superior			Inferior	Superior	
Sex	0.431	0.233	0.798	0.007	1.904	0.506	7.162	0.341
Age	1.171	1.118	1.226	0.000	1.087	1.006	1.174	0.035
BMI	0.884	0.823	0.950	0.001	1.368	0.391	4.786	0.624
Study Level	0.171	0.097	0.300	0.000	0.334	0.144	0.775	0.011
Economic Level	0.617	0.331	1.152	0.130	NS	NS	NS	NS
Diabetes Mellitus	3.185	1.472	6.891	0.003	3.973	1.048	15.054	0.042
Cardiovascular Disease	1.806	1.010	3.228	0.046	0.453	0.140	1.468	0.187
Anxiety	0.400	0.093	1.725	0.219	NS	NS	NS	NS
Depression	2.323	0.936	5.765	0.069	NS	NS	NS	NS
History of Dementia	1.759	0.975	3.171	0.060	NS	NS	NS	NS
Physical Activity	0.086	0.043	0.170	0.000	0.147	0.045	0.479	0.001
Number of falls in the last year	1.808	1.312	2.491	0.000	1.409	1.054	1.885	0.021
Healthy Diet	0.263	0.136	0.509	0.000	0.560	0.175	1.787	0.327
Number of visits to physician	0.790	0.690	0.906	0.001	0.621	0.481	0.802	0.000
Number of Drugs	1.416	1.250	1.603	0.000	1.437	1.154	1.790	0.001
Sleep Disturbances	1.647	0.892	3.040	0.111	NS	NS	NS	NS

Abbreviations: NS: Non-Significance (not included in the multivariate analysis); 95% C.I.: 95% Confidence Interval; *p*: *p*-value; OR: Odds Ratio; BMI: Body Mass Index.

**Table 3:** Univariate and multivariate logistic regression to analyze the factors related to sleep disturbances in subjects with Alzheimer’s disease.

Variable	OR	Univariate Analysis		*p*	OR	Multivariate Analysis		*p*
		95% C.I.				95% C.I.		
		Inferior	Superior			Inferior	Superior	
Sex	1.687	0.714	3.989	0.233	NS	NS	NS	NS
Age	0.969	0.920	1.021	0.243	NS	NS	NS	NS
BMI	1.087	0.997	1.184	0.057	NS	NS	NS	NS
Study Level	0.645	0.340	1.226	0.181	NS	NS	NS	NS
Economic Level	0.894	0.455	1.757	0.744	NS	NS	NS	NS
Diabetes Mellitus	0.647	0.284	1.476	0.301	NS	NS	NS	NS
Cardiovascular Disease	0.522	0.242	1.127	0.098	NS	NS	NS	NS
Anxiety	3.045	0.268	34.607	0.369	NS	NS	NS	NS
Depression	0.549	0.211	1.424	0.217	NS	NS	NS	NS
History of Dementia	1.647	0.774	3.501	0.195	NS	NS	NS	NS
Physical Activity	0.583	0.219	1.555	0.281	NS	NS	NS	NS
Number of falls in the last year	2.210	1.461	3.342	0.000	2.145	1.369	3.361	0.001
Healthy Diet	0.815	0.385	1.724	0.592	NS	NS	NS	NS
Number of visits to physician	1.273	1.025	1.580	0.029	1.173	0.916	1.503	0.206
Number of Drugs	0.948	0.842	1.068	0.378	NS	NS	NS	NS

Abbreviations: NS: Non-Significance (not included in the multivariate analysis); 95% C.I.: 95% Confidence Interval; *p*: *p*-value; OR: Odds Ratio; BMI: Body Mass Index.

**Table 4:** Univariate and multivariate linear regression to analyze the factors related to the number of falls in subjects with Alzheimer’s disease.

Variable	β	Univariate Analysis		*p*	β	Multivariate Analysis		*p*
		95% C.I.				95% C.I.		
		Inferior	Superior			Inferior	Superior	
Sex	0.175	−0.036	1.400	0.062	NS	NS	NS	NS
Age	−0.073	−0.061	0.027	0.445	NS	NS	NS	NS
BMI	0.101	−0.032	0.106	0.284	NS	NS	NS	NS
Study Level	−0.083	−0.745	0.288	0.382	NS	NS	NS	NS
Economic Level	−0.041	−0.691	0.440	0.661	NS	NS	NS	NS
Diabetes Mellitus	0.022	−0.596	0.754	0.817	NS	NS	NS	NS
Cardiovascular Disease	−0.167	−1.209	0.062	0.077	NS	NS	NS	NS
Anxiety	0.068	−1.244	2.668	0.472	NS	NS	NS	NS
Depression	0.169	−0.065	1.530	0.071	NS	NS	NS	NS
History of Dementia	0.218	0.118	1.343	0.020	0.212	0.149	1.270	0.014
Physical Activity	0.104	−0.345	1.210	0.273	NS	NS	NS	NS
Healthy Diet	0.075	−0.373	0.878	0.426	NS	NS	NS	NS
Number of visits to physician	0.355	0.159	0.468	0.000	0.319	0.132	0.432	0.000
Number of Drugs	−0.045	−0.121	0.074	0.632	NS	NS	NS	NS
Sleep Disturbances	0.329	0.521	1.729	0.000	0.235	0.219	1.386	0.007

Abbreviations: NS: Non-Significance (not included in the multivariate analysis); 95% C.I.: 95% Confidence Interval; *p*: *p*-value; β: Beta-value; BMI: Body Mass Index.

## Data Availability

The data presented in this study are available on request from the corresponding author. The data are not publicly available because, due to the sensitive nature of the questions asked in this study, participants were assured raw data would remain confidential and would not be shared.

## Supplementary Materials

The following supporting information can be downloaded at: https://www.mdpi.com/article/10.3390/healthcare11212852/s1.
