# Supplementary material for: Falls and Sleep Disorders in Spanish Alzheimer’s Disease in Nursing Homes: An Observational Study
Source: Healthcare (Basel). 2023 Oct 30;11(21):2852. doi: 10.3390/healthcare11212852 (PMC10649919; doi:10.3390/healthcare11212852)
Supplement: Supplementary file 1 [file healthcare-11-02852-s001.zip › healthcare-2655809-SI.pdf]

Observational study: survey

Hello, in this questionnaire you must answer the following questions about the history and related habits that you have had throughout your life, to help to identify possible risk factors that predispose or protect against Alzheimer's disease. It will take you about 10 minutes. Thank you very much for your cooperation.

To do this, you must be at least 65 years old and not suffer from any neurodegenerative disease. You can fill out the survey representing a family member who is not familiar with new technologies, as long as they give their consent.

Read the questions carefully and answer as truthfully as possible.

1. Age

____

2. Gender

Marca solo un óvalo.

- ◯ Man
- ◯ Woman
- ◯ Non-binary

3. Place of residence

Marca solo un óvalo.

- ◯ Habitual or family address
- ◯ Nursing home

4. Civil status

Marca solo un óvalo.

- Single
- Married
- Widow/ed

5. Profession

____

6. Any neurodegenerative disorder in your family? (Dementia, Parkinson, Alzheimer's, multiple sclerosis, Amyotrophic Lateral Sclerosis...)

Marca solo un óvalo.

- Yes
- No

7. Study level

Marca solo un óvalo.

- None
- School
- High school
- University or superior

8. Have you been a physically active person throughout your life (at least 150 minutes/week of moderate physical activity)?

Marca solo un óvalo.

- Yes
- No

9. How many falls have you suffered during the last year? Indicate it with number

____

10. Did or do you have harmful habits such as tobacco, alcohol or drug use?

Marca solo un óvalo.

- No
- Alcohol
- Tobacco
- Drugs
- More than one
- More than two

11. Do you think you have had a healthy diet throughout your life?

Marca solo un óvalo.

- Yes
- No

12. Please indicate your approximate weight (kilograms)

____

13. Please indicate your approximate height (meters)

____

14. How many times have you gone to the doctor in the last year? Whether by telephone or in person. Indicate it with numbers

____

15. How many drugs do you take daily? Write the drugs and their daily dosage

____

16. Do you need a caregiver all the time?

Marca solo un óvalo.

- Yes
- No

17. Do you have sleep problems? For example: Do you have sleep problems? For example: insomnia, excessive sleepiness, sleep apnea, or fractionated sleep ...

Marca solo un óvalo.

- Yes
- No

18. What economic level do you estimate you have had throughout your life?

Marca solo un óvalo.

- Low
- Medium
- High

19. Have you suffered continuous stress (family and/or work) throughout your life or has there been a traumatic event that has triggered great suffering?

Marca solo un óvalo.

- Yes
- No

20. Have you provided a copy of your medical history issued by the national health system? Please, if still not contact the responsible researchers by phone or at the email that appears in the informed consent

Marca solo un óvalo.

- Yes
- No

Este contenido no ha sido creado ni aprobado por Google.

Google Formularios
